# Development and Integration of DOPS as Formative Tests in Head and Neck Ultrasound Education: Proof of Concept Study for Exploration of Perceptions

**DOI:** 10.3390/diagnostics13040661

**Published:** 2023-02-10

**Authors:** Johannes Matthias Weimer, Maximilian Rink, Lukas Müller, Klaus Dirks, Carlotta Ille, Alessandro Bozzato, Christoph Sproll, Andreas Michael Weimer, Christian Neubert, Holger Buggenhagen, Benjamin Philipp Ernst, Luisa Symeou, Liv Annebritt Lorenz, Anke Hollinderbäumer, Julian Künzel

**Affiliations:** 1 Rudolf Frey Teaching Department, Mainz University Hospital, 55131 Mainz, Germany; 2 Department of Otorhinolaryngology, Regensburg University Hospital, 93053 Regensburg, Germany; 3 Department of Diagnostic and Interventional Radiology, Mainz University Hospital, 55131 Mainz, Germany; 4 Department of Gastroenterology and Internal Medicine, Rems-Murr-Klinikum, 71364 Winnenden, Germany; 5 Department of Otorhinolaryngology, University of Saarland, 66123 Homburg, Germany; 6 Department of Oral and Maxillofacial Surgery, University Hospital Düsseldorf, Heinrich-Heine-University Düsseldorf, 40225 Düsseldorf, Germany; 7 Department of Orthopedics, Trauma Surgery, and Spinal Cord Injury, Heidelberg University Hospital, 69120 Heidelberg, Germany; 8 Department of Otorhinolaryngology, University Medical Center Bonn (UKB), 53127 Bonn, Germany; 9 Department of Radiooncology and Radiotherapy, Mainz University Hospital, 55131 Mainz, Germany

**Keywords:** head and neck ultrasonography, direct observation of procedural skills (DOPS), ultrasound training, quality, teaching, diagnostic

## Abstract

In Germany, progress assessments in head and neck ultrasonography training have been carried out mainly theoretically and lack standardisation. Thus, quality assurance and comparisons between certified courses from various course providers are difficult. This study aimed to develop and integrate a direct observation of procedural skills (DOPS) in head and neck ultrasound education and explore the perceptions of both participants and examiners. Five DOPS tests oriented towards assessing basic skills were developed for certified head and neck ultrasound courses on national standards. DOPS tests were completed by 76 participants from basic and advanced ultrasound courses (*n* = 168 documented DOPS tests) and evaluated using a 7-point Likert scale. Ten examiners performed and evaluated the DOPS after detailed training. The variables of “general aspects” (6.0 Scale Points (SP) vs. 5.9 SP; *p* = 0.71), “test atmosphere” (6.3 SP vs. 6.4 SP; *p* = 0.92), and “test task setting” (6.2 SP vs. 5.9 SP; *p* = 0.12) were positively evaluated by all participants and examiners. There were no significant differences between a basic and advanced course in relation to the overall results of DOPS tests (*p* = 0.81). Regardless of the courses, there were significant differences in the total number of points achieved between individual DOPS tests. DOPS tests are accepted by participants and examiners as an assessment tool in head and neck ultrasound education. In view of the trend toward “competence-based” teaching, this type of test format should be applied and validated in the future.

## 1. Background

Training in ultrasonography is increasingly becoming an essential part of medical education in almost all specialities, both nationally and internationally [1], and increased attention is being given to it in human medicine courses [2,3]. Ways of extending and improving training standards for medical ultrasounds are also a matter of lively debate in the specialist literature [4]. In addition to the practical experience gained in everyday clinical work, ultrasound courses are an important component of training in diagnostic ultrasound. These courses are based on the curricula developed by the relevant professional societies [5] and on the requirements set out by the Association of Statutory Health Insurance Physicians in Germany (Kassenärztliche Vereinigung) and similar national institutions [6]. In the context of such courses, efforts to obtain certifications include the central question of how to examine the success of learning theoretical and practical topics and how to ensure this and document it in a standardised way [7]. Although the Association of Statutory Health Insurance Physicians requires colloquia in some areas to review ultrasound skills, more specific requirements are not yet available [6]. New international recommendations also deal with competence assessment in head and neck ultrasound training [8].

Tests are used for quality control and to obtain evidence of knowledge, skills, and competence. The test results can be presented either using a summated score [9], or formatively. In the latter case, the focus is on checking and communicating learning progress and defining further learning objectives [10]. On the basis of Miller’s knowledge pyramid [11], different test formats can be assigned to different competence levels (Supplementary Figure S1).

If these conclusions are applied to ultrasound courses in the field of medical training, the aim of such courses must be to provide professional teaching of diagnostic ultrasound skills at the highest level, “DOES”, which would be the level for testing ultrasound skills. A structured method of observation and evaluation during everyday clinical work is required for this purpose. The widely used objective structured clinical examination (OSCE) method assesses practical clinical skills using the example of standardised test situations [12]. Consequently, this only allows checking of the “shows how” level.

Test formats that are used to test the “DOES” level include the mini-clinical evaluation exercise (mini-CEX) and a direct observation of procedural skills (DOPS) [13,14,15]. Examiners use checklists to assess realistic work situations or real doctor–patient interactions. Each observation includes constructive, standardised feedback, with suggestions for further improvement. In contrast to the classic OSCE test, DOPS tests can be incorporated into the course sequence flexibly and easily. They can also provide a kind of horizon of expectations for teachers and apprentices and contribute to an improvement in the quality of teaching [16,17].

### 1.1. Test Formats in Ultrasound Training

In the field of ultrasound training, the implementation of written assessments of learning outcomes that can be used before, during, and after the course have been described in the literature for measuring theoretical competence [18]. These purely theoretical knowledge tests are often based on the professional societies’ course curricula [6], but different sources claim a competence-based assessment of skills [8,18]. However, there are no uniform content specifications here in relation to scope, question type, or question structure. In the context of ultrasound courses, evaluating learning success without taking practical competence into account is only of limited value.

Various methods have already been developed for assessing (ultrasound) skills as objectively as possible [14,18,19,20,21], including OSCEs [20] and DOPS tests [14,15,16,17,22], as well as the objective structured assessment of ultrasound skills scale (OSAUS) [21,22]. In the areas of abdominal ultrasound and emergency ultrasonography, structured tests using OSCEs as well as the OSAUS are available [19,20]. Todsen et. al. tested the use of the OSAUS to assess the “focused head and neck ultrasound” competence of surgeons [20].

### 1.2. DOPS Tests in Otorhinolaryngology

DOPS tests are already used internationally in the field of otorhinolaryngology as a format for testing and simultaneously teaching clinical skills during educational and training courses [23,24,25,26]. Significant improvements in skills have been reported, particularly during the initial years of residency training [24,26]. However, testing of ultrasound competence was not included in the DOPS described. The aim of this proof-of-concept study was to develop the first DOPS tests for head and neck ultrasound and to describe their integration into sonography courses. In addition, the participants’ and examiners’ perceptions and acceptance of the DOPS will be evaluated.

## 2. Methods

### 2.1. Head Neck Ultrasound DOPS Test Development

**Figure 1:**
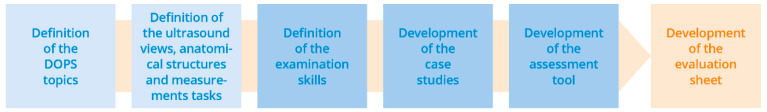
Development steps involved in establishing and evaluating direct observation of procedural skills (DOPS) tests in head and neck ultrasonography.

The basis for the design of the DOPS tests was the content of the basic ultrasonography catalogue published by the Head and Neck Section of the German Society for Ultrasound in Medicine (Deutsche Gesellschaft für Ultraschall in der Medizin, DEGUM) and current specialist articles on continuing medical education (CME) [5,18,27]. The development of the DOPS and the associated evaluation tools was supported by experts from various disciplines (otolaryngology, radiology, neurology, neurosurgery, general surgery, and medical education). The individual development steps are indicated in Figure 1.

A total of five subject areas were defined for the organ/structure examination, with the corresponding orientation sections and landmarks, as well as the ultrasound-specific content of the corresponding DOPS and possible measurements (Table 1).

A catalogue of requirements was drawn up for DOPS participants, which was used to check their examination-related abilities and examination techniques (“skills”) (depicted in the Section 3). For this purpose, assessment areas were defined and a maximum number of points (37 points) was determined [28] also with a view to the OSCE sheets by Hofer et al. [20]. The points are distributed between “patient communication” (6 points), “transducer handling” (6 points), “image optimization” (2 points), “examination performance” (6 points), “measurement and assessment” (6 points), “image explanation and documentation” (5 points), and “overall performance” (5 points).

The DOPS test was developed through a process of expert consensus, taking levels of difficulty into account that were as comparable as possible. In this study, the level of difficulty of the DOPS tests was deliberately oriented towards a basic level of head and neck ultrasound skills. In addition, typical clinical case vignettes/settings were developed for each DOPS test and a time scheme involving a test time of 10 min (8 min for test performance and 2 min for feedback) was established (an example is shown in Supplementary Figure S2). Appropriate task sheets were then prepared for the examiners and examinees (Supplementary Figure S2).

### 2.2. Evaluation Tools for the Exploration of Perceptions and Attitudes

Examiner-specific and participant-specific questionnaires were developed to evaluate the perceptions and attitudes of the DOPS tests that were conducted. The questionnaire items were evaluable on a 7-point Likert scale, with options ranging from “is not at all correct” (=1) to “is completely correct” (=7). Both questionnaires included free-text fields for comments related to “positive and negative aspects”. For the examiners, another free-text field was added to inquire about “factors influencing examiners”. The construction of the evaluation questionnaires was based on the Trier Teaching Assessment Inventory [29] and studies by Pierre et al. [30] and Weisser et al. [31]. The evaluation form includes a total of 29 items for participants and 28 items for examiners. The categories asked about were related to “DOPS/test in general” (D), “test atmosphere” (A), “test tasks” (T), “participant satisfaction” (P), and “examiner satisfaction” (E), with a total of 23 items identical in both forms (Table 2).

### 2.3. Test Procedure, Participants, and Examiners

To evaluate the development, perceptions, and attitudes of the DOPS tests, they were used at a DEGUM-certified course in basic and advanced head and neck ultrasonography held in 2021. The participants were the examinees, and the examiners were selected lecturers and tutors (Supplementary Tables S1 and S2).

The courses each comprised 16 teaching units on at least 2 days, with theory in the advanced course (8 units) being taught in the form of a webinar prior to the practical exercises. The practical exercises (8 units) were conducted by all of the participants in rotation. During the courses, the participants completed at least one DOPS test themselves and were present for the running of the other DOPS tests in their small group. This proof-of-concept investigation did not include individual testing or blinding. The examiners selected DOPS tests thematically according to their assigned practice station.

The examiners were instructed on how to conduct the DOPS tests. This included a detailed discussion of case vignettes, the evaluation form, and the test procedure.

### 2.4. Statistical Analysis

All statistical analyses and graphics were conducted using R studio (RStudio Team. RStudio: Integrated Development for R. 2020) with R 4.0.3 (R Foundation for Statistical Computing. A Language and Environment for Statistical Computing). Binary and categorical baseline parameters are expressed as absolute numbers and percentages. Continuous data are expressed as median and interquartile range (IQR), or as mean and standard deviation (SD). Categorical parameters were compared using Fisher’s exact test, and continuous parameters using the Mann–Whitney test. *p* values < 0.05 were considered statistically significant.

## 3. Results

### 3.1. Sample Description

A total of 76 participants and 10 examiners participated in the study. Most of the participants were attending the basic course (75.0%), were ear, nose, and throat specialists (residents in otorhinolaryngology) (80.3%), had not previously attended an ultrasound course (65.8%), and had performed fewer than 100 independent examinations (64.5%). All of the examiners had experience or certification in ultrasound training (Supplementary Tables S1 and S2).

### 3.2. Results of the Evaluation of DOPS

**Figure 2:**
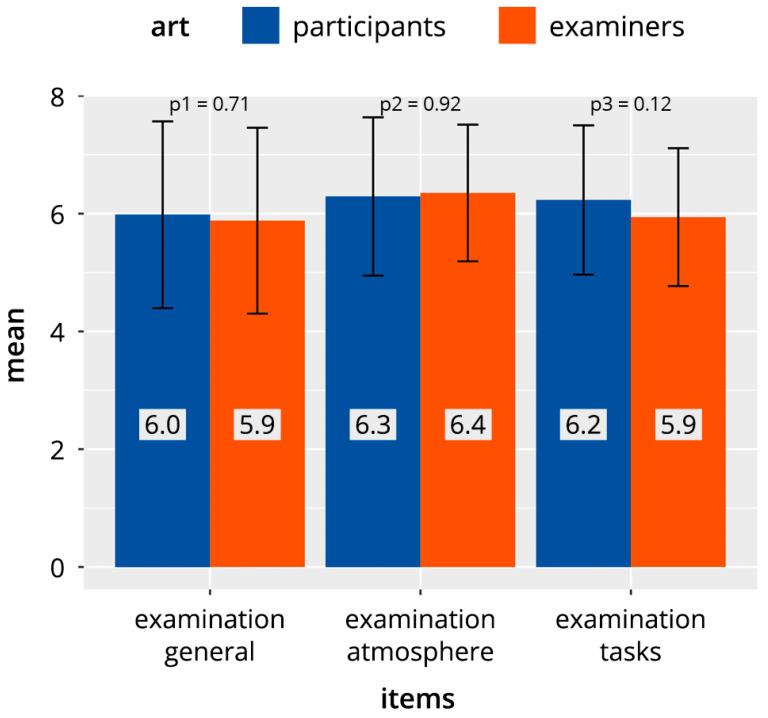
Comparison of the higher-level common item categories in direct observation of procedural skills (DOPS) tests in participants and examiners.

Figure 2 shows the cumulative evaluation results of all identical items for the three categories “DOPS test in general” (D), “test atmosphere” (A), and “test tasks” (P), which were answered by both examiners and participants. The mean values for the two groups showed values in the range of 5.9–6.4 scale points, with no significant differences between examiners and participants.

**Figure 3:**
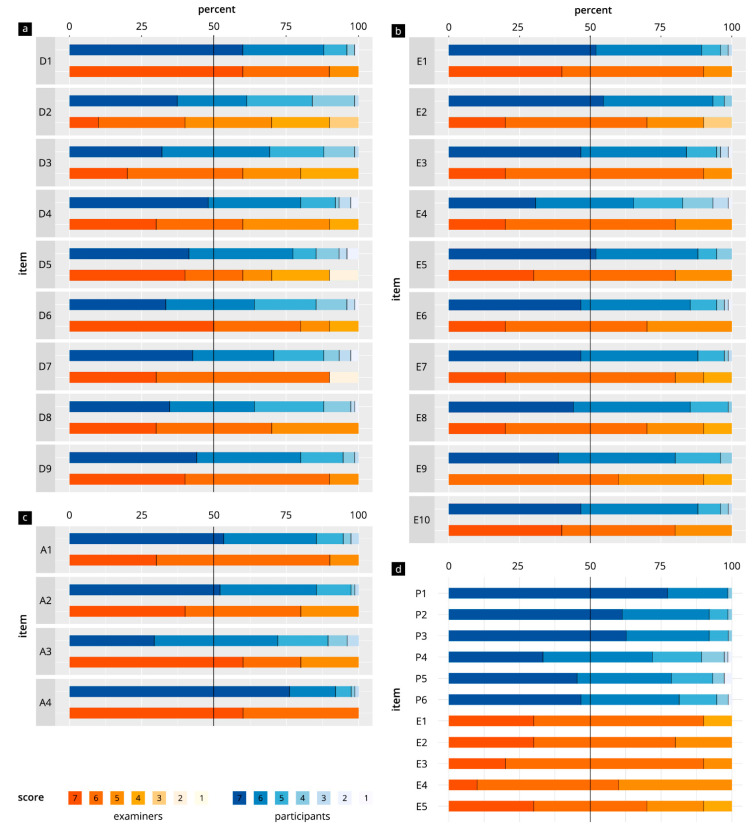
Comparison of items in direct observation of procedural skills (DOPS) tests in participants and examiners. (**a**) General aspects of the DOPS (D1–D9). (**b**) Test atmosphere (A1–A4). (**c**) Test tasks (T1–T10). (**d**) Specific items for the participants (P1–P6) and examiners (E1–E5).

Figure 3 and Table 2 show the evaluation results for all items in the categories “DOPS test in general” (D1–D9), “test atmosphere” (A1–A4), “test task” (T1–T10), “participant-specific items”, and “examiner-specific items”. In the participant group, the results were in the range of 5.7–6.8 points. The evaluation results for the examiner items were in the range of 5.1–6.6 points. There were significant differences in the evaluations for the items “feasibility of the tasks with sufficient preparation” (*p* = 0.01) and “measurements/assessment tasks” (*p* = 0.02), with the examiners giving both of these items lower scores. With regard to participant-specific and examiner-specific items, “adequate examiner communication” (T1) and “structure of the evaluation sheet” (E3) were evaluated best.

### 3.3. Free-Text Comments

The majority of the 29 participant comments mentioned a “pleasant test atmosphere” and the “fair and good examination of practical learning success”. Points of criticism were a “perceived heterogeneity of the examiner guidance” and “inconsistent feedback”. The participants also expressed a desire for DOPS tests on other topics, such as the larynx. The free-text comment option was used by three of the ten examiners, requesting “an increase in the difficulty of the test” and “additional assessment criteria”.

### 3.4. Results of the DOPS Carried Out

**Figure 4:**
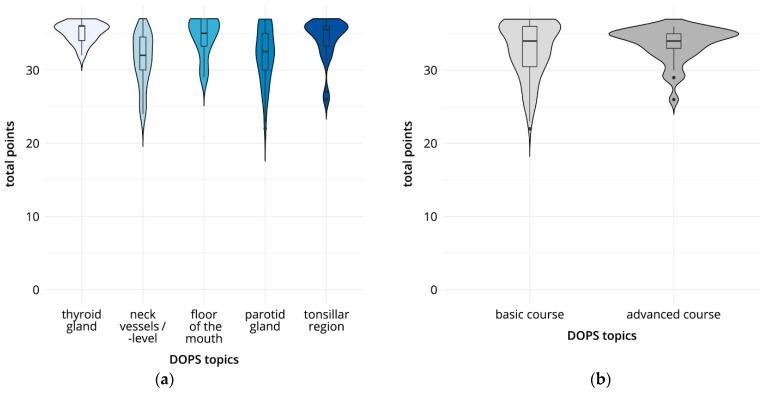
Direct observation of procedural skills (DOPS) tests: results comparing total points (**a**) and course format (**b**). (**a**) Total points for the five DOPS tests. (**b**) Total points in the basic and advanced course formats.

Of the total 168 DOPS tests documented, 135 were performed in the basic course and 33 in the advanced course. In the overall analysis, the results ranged from 31.4 points for the DOPS test on the topic “cervical vessels/cervical level” to 35.2 points for the DOPS test on the topic “thyroid gland,” out of a total of 37 possible evaluation points (Figure 4, Table 3). There were significant differences in the total scores achieved between DOPS I (“thyroid”) and DOPS II (“cervical vessels/cervical level”) (*p* < 0.01); between DOPS I (“thyroid”) and DOPS IV (“parotid gland”) (*p* < 0.01); between DOPS II (“cervical vessels/cervical level”) and DOPS III (“floor of the mouth”) (*p* < 0.01); and between DOPS II (“cervical vessels/cervical level”) and DOPS V (“submandibular fossa”) (*p* < 0.01). Evaluation of the subcategory “image optimization” was significantly poorer in DOPS IV than in DOPS V. The “examination procedure” was rated significantly lower (*p* < 0.01) in DOPS II than in DOPS III. The “measurements” performed were significantly better (*p* < 0.01) in DOPS I and DOPS V than in DOPS II and DOPS IV. In addition, overall performance was rated significantly better (*p* < 0.01) in DOPS I and DOPS III than in DOPS II. Figure 4 also shows the mean scores for all DOPS tests for the participants in the basic course (mean 32.8, SD 3.7) and advanced course (mean 33.6, SD 2.4). The comparison did not show any statistically significant differences. The narrower distribution range for the results of the participants in the advanced course is notable.

## 4. Discussion

This study is the first to evaluate ultrasound DOPS testing in head and neck ultrasonography training. The data show that the concept and implementation of DOPS testing were accepted by the participants and examiners. In addition, the DOPS tests made it possible for previously defined practical learning objectives to be verified and demonstrably achieved through educational testing. The results of this ‘proof of concept’ study are encouraging for the development of practical test formats for ultrasound training courses and their further validation.

Although practical tests are already well established in student ultrasound training courses [14,18], only occasional attempts have so far been made to use them in ultrasound during residency training [15,19]. However, comprehensive and structured testing of practical skills is required [18]. In the DEGUM courses on head and neck ultrasonography in particular, only attendance and optional passing of a test on theoretical content are currently required for successful participation [5]. Quality assurance is mainly left to the degree of personal commitment by the course instructors. Institutions such as the Association of Statutory Health Insurance Physicians (Kassenärztliche Vereinigung) claim colloquia for testing skills and knowledge mainly to approve reimbursement for ultrasound examinations, but the content of these does not have a uniform structure.

The results of the present study show that DOPS testing is quite feasible, simple to perform, and provides largely objective, practical quality control at the physician level. Transferring the findings of ultrasound training to other medical specialties seems possible. Interestingly, only significant differences were found in the rating of the items “feasibility of the tasks with sufficient preparation” and “measurements/assessment tasks”. Although both participants and examiners rated the items in high scale ranges (≥5.5 points), the rating by the participants was significantly higher. This could be explained by the higher qualification of the examiners in terms of standardised examination procedures and clinical experience and should be taken as an opportunity to further modify the examination forms in the future.

As OSCEs are usually structured in the form of a circuit with several stations per participant, a large investment of time and resources is required to implement the courses. In contrast, DOPS testing can be included in the course sequence repeatedly in the form of individual tests (and could potentially also be included in everyday clinical work). In our view, this represents a significant advantage for DOPS testing, particularly in the setting of course formats involving several days. Structured DOPS tests can also be used as an educational tool during practical exercises [17,25] and can help to improve training quality when conducted repeatedly [32].

Initial efforts to test practical skills using DOPS tests have already been made [24,25,26]. The time frame selected in the present study (10 min per DOPS test) has been used in this method [24,25] and was well evaluated by both examiners and examinees. In accordance with our results, DOPS tests have been investigated by other research groups as a useful and effective technique for providing continuing training [23,24] and have been positively evaluated [23,24,32]. The present results show that DOPS testing can help participants identify their strengths and weaknesses while at the same time increasing their motivation to further improve their skills, as has also been shown in other medical specialities [23].

The use of DOPS testing in ultrasound courses thus has benefits for everyone involved. However, additional practical testing within the framework of courses requires more time and staff [15,20,24,25]. Modern course models—using digital preparation items, for example—provide an opportunity to make individual aspects of the theoretical content available before the course starts. Greater emphasis can then be given to the development of practical skills during the attendance period.

Earlier studies have shown that DOPS tests are capable of reflecting learning progress to some extent [15,23,24,26,32]. In this explorative study, no significant differences were found between participants in the basic course and those in the advanced course relative to the mean total scores achieved. The only notable difference was a wider distribution range of the results among participants in the basic course. Among advanced examinees, using DOPS tests did not lead to greater discriminatory power, which has also been observed in other publications [24,26]. The results confirm the designed difficulty level of the DOPS tests used in the present study, which has been explicitly oriented toward the basic course level. This aspect was also reflected in the examiners’ free-text comments, requesting a greater difficulty level. Useful approaches to ensuring that advanced examiners’ competence can also be evaluated appropriately might include extending the tasks featured in existing DOPS tests (e.g., with additional use of colour Doppler), compiling additional DOPS tests with greater difficulty levels (e.g., laryngeal ultrasound or ultrasound-guided puncture), and/or using DOPS in everyday clinical work. This should be investigated and validated in further research. The inclusion of clinical decision making (establishing an indication, carrying out further diagnosis, and therapeutic implications) would also be useful. Decision making is already tested in OSAUS assessments [21,22], and this should be transferred to an optimised DOPS test concept in the examinations in the clinical routine or setting. The point weighting of the OSAUS scale (each examination item has the same maximum score, e.g., for “indication naming” and “systematic examination (including measurements)”) is, in our view, not in proportion to the practically oriented learning objectives defined for a basic course and should, therefore, be adapted in the sense of the DOPS examinations.

A detailed examination of the present findings shows that performance in DOPS II tended to be poorer. One possible explanation for this might be that the assessment of cervical vessels only plays a subordinate role in everyday clinical practice in otorhinolaryngology so far (which was the specialty of most participants in this study), so that the participants had correspondingly less previous experience. In the future, better differentiation by creating separate DOPS tests for the topics “cervical level” and “cervical vessels” would be desirable.

A follow-up of individual participants is not yet possible because this was out of scope of the present study. Future studies should aim to investigate structured and longitudinal practical performance in the setting of ultrasound training courses. This would be possible within the DEGUM course system if the participants agree. Correlating the results with the participants’ individual practical ultrasound experience would also be an interesting aspect [33,34].

In addition to certified course systems, structured, uniform DOPS tests should also be used increasingly to provide instructors with qualifications. Initial approaches of this type by the specialist associations already exist in the context of the examination for DEGUM level II in anesthesiology [35].

In order to meet the demand for better and uniform quality assurance in the setting of certified ultrasound training formats, practical examinations should be jointly developed and accredited by the certifying institutions in collaboration with educational experts [7]. It should also be noted that current “train the trainer” approaches should increasingly include the creation and implementation of practical and theoretical examinations. Instruction for examiners is naturally one of the most important building blocks for the qualified implementation of DOPS testing [15]. More intensive examiner training is also planned for the further development of DOPS testing in our own course concept in the future.

Digital test formats [36,37] could be developed to facilitate the documentation of test performance and to allow easier tracking of increasing theoretical and practical competence among the participants.

In principle, the aim should be to standardise the currently coexisting formats “OSCE”, “DOPS”, and “OSAUS” in the field of head and neck ultrasound and relevant subjects. A uniform international standard for the assessment of practical competence (independent of the course provider and format) would be an idealistic but important quality criterion. A prerequisite for that would be the extension of existing quality assurance requirements for ultrasound courses to include mandatory practical examinations and the appropriate content requirements. In the area of head and neck ultrasound, the current training recommendations of the EFSUMB can provide good orientation [7]. Specific DOPS or OSAUS (e.g., performance and interpretation of a DOPS for contrast medium sonography of an enlarged lymph node for level 3) should be developed and validated for the respective competence levels. These can then be used to classify individual skills.

### Limitations

Since the participants in this study only provided information about their identity on a voluntary basis, adequate longitudinal observation of progress in their performance was not possible. Similarly, the selection of the examiners was not homogeneous in relation to the level of experience, and instruction for the examiners has not yet been standardised. In addition, the individual DOPS tests were carried out with varying frequency, as the examiners were also able to select the DOPS according to their personal preferences. The DOPS tests were performed within a small group, without blinding of the other group members so some learning and habituation effects might therefore have influenced the results. Another weakness of this study is the fact that the DOPS tests have not yet been used in courses taught by different providers/course instructors. In this monocentric design, selection bias in the evaluation of the DOPS tests cannot be ruled out. In addition, no validation was carried out in this study because the group of participants was too small and homogeneous.

## 5. Conclusions

Structured, clearly arranged formats for quality assessment of clinical skills such as ultrasonography represent a useful instrument both during training courses and in clinical continuing education. Due to the trend toward “competence-based” training, this type of examination format should be further applied, evaluated, and validated in the future.

## Figures and Tables

**Table 1:** Direct observation of procedural skills (DOPS) test topics in head and neck ultrasonography, with learning objectives and landmarks.

Topics	Content	Landmark Structures in Orientation Sections	Assessment
**Thyroid gland**			
DOPS I	Assessment of thyroid, trachea, esophagus	Thyroid, trachea, esophagus, common carotid artery; internal jugular vein, sternocleidomastoid, omohyoid, spinal column	Thyroid gland volume
**Cervical vessels and cervical level**			
DOPS II	Assessment of cervical vessels and cervical level	Common carotid artery, internal carotid artery, external carotid artery, internal jugular vein. sternocleidomastoid, trapezius, omohyoid, posterior belly of digastric muscle	Intima–media thickness, arterial flow profiles
**Floor of the mouth**			
DOPS III	Assessment of the floor of the mouth and larynx	Anterior belly of digastric muscle, mylohyoid, geniohyoid, sublingual gland, tongue, vallecula of hyoid bone, thyroid cartilage, arytenoid cartilage	Measurement of the sublingual gland, with bilateral comparison
**Parotid gland**			
DOPS IV	Assessment of the parotid gland	Sternocleidomastoid, masseter, posterior belly of digastric muscle, retromandibular vein, mandible	Visualisation of the retromandibular vein in transverse and sagittal section
**Submandibular fossa**			
DOPS V	Assessment of the submandibular fossa	Submandibular gland, tonsils, tongue, mylohyoid	Measurement of the tonsils, with bilateral comparison

**Table 2:** Evaluation results of identical items between participants and examiners.

Item	Description	Participants		Examiners		P
		Mean	SD	Mean	SD	
**DOPS—general aspects**						
D1	Practical skills review	6.4	0.9	6.5	0.7	0.95
D2	Review of theoretical knowledge	5.8	1.1	5.1	1.2	0.08
D3	Differentiated performance assessment	5.9	1.0	5.6	1.1	0.40
D4	Good educational tool	6.1	1.2	5.8	1.0	0.21
D5	Use in any practical ultrasound course	5.9	1.3	5.5	1.7	0.52
D6	Clear structure	5.8	1.2	6.2	1.0	0.26
D7	Positive learning effect	5.9	1.3	5.9	1.4	0.98
D8	Fair performance evaluation	5.8	1.1	6.0	0.8	0.81
D9	Clinically relevant content and examination procedures	6.2	0.9	6.3	0.7	0.88
**Test atmosphere**						
A1	Appropriate use of time	6.3	0.9	6.2	0.6	0.33
A2	Clear wording of instructions and notes	6.3	0.8	6.2	0.8	0.5
A3	Sufficiently prepared for the test	5.9	1.0	6.4	0.8	0.11
A4	Relaxed test atmosphere	6.6	0.8	6.6	0.5	0.40
**Test tasks**						
T1	Understandable tasks	6.3	0.8	6.3	0.7	0.58
T2	Feasibility of tasks with sufficient preparation	6.4	0.7	5.6	1.4	0.01
T3	Reasonable level of difficulty	6.2	1.0	6.1	0.6	0.30
T4	DOPS I–V with comparable level of difficulty	5.7	1.2	6.0	0.7	0.73
T5	Comprehensible expectations	6.4	0.8	6.1	0.7	0.22
T6	Clear task definition	6.2	1.0	5.9	0.7	0.11
T7	Ultrasound views	6.3	0.8	5.9	0.9	0.12
T8	Case studies	6.3	0.8	5.8	0.9	0.10
T9	Measurements/assessment tasks	6.2	0.8	5.5	0.7	0.02
T10	Demonstration tasks	6.3	0.8	6.2	0.8	0.061
**Special items for participants**						
P1	Appropriate examiner communication	6.8	0.5			
P2	Patient consideration	6.5	0.7			
P3	Communication with patient	6.5	0.7			
P4	Personal strengths and weaknesses	5.9	1.1			
P5	Motivation to further improve/deepen skills	6.1	1.1			
P6	Role fulfilment of participant	6.2	1.0			
**Special items for the examiners**						
E1	Comfortable in the role of the examiner			6.1	0.9	
E2	Evaluation sheet structure			6.1	0.7	
E3	Content evaluation sheet			6.1	0.6	
E4	Clarity of the evaluation scheme			5.7	0.7	
E5	Evaluation scheme for examination procedures			5.9	1.0	

**Table 3:** Results of the direct observation of procedural skills (DOPS) I–V tests conducted.

	DOPS No.				
	I	II	III	IV	V
Subject	Thyroid	Cervical Vessels/Level	Floor of the Mouth	Parotid	Tonsillar Region
Total	17	35	22	60	34
Basic course	12	22	17	55	29
Advanced course	5	13	5	5	5
Total score, mean (SD)	35.2 (1.4)	31.4 (3.6)	34.6 (2.7)	32.0 (3.8)	34.2 (2.9)
Subcategories, mean (SD)					
Patient management	5.6 (0.6)	5.7 (0.7)	5.9 (0.4)	5.7 (0.9)	5.8 (0.7)
Transducer handling	6.0 (0.0)	5.4 (1.1)	5.9 (0.2)	5.5 (0.8)	5.9 (0.5)
Image optimisation	1.7 (0.5)	1.7 (0.5)	1.9 (0.4)	1.5 (0.5)	1.9 (0.4)
Examination course	5.6 (0.6)	4.9 (1.0)	5.7 (0.6)	5.1 (1.0)	5.4 (1.3)
Measurements	5.9 (0.5)	4.0 (1.8)	4.9 (1.5)	4.5 (1.5)	5.3 (1.1)
Image explanation	5.0 (0.0)	5.0 (0.0)	5.0 (0.2)	5.0 (0.12)	5.0 (0.0)
Overall impression	5.3 (0.6)	4.7 (0.7)	5.3 (0.8)	4.9 (1.0)	4.9 (0.5)

## Data Availability

Data cannot be shared publicly because of institutional and national data policy restrictions imposed by the Ethics committee since the data contain potentially identifying study participants’ information. Data are available upon request from the Johannes Gutenberg University Mainz Medical Center (contact via weimer@uni-mainz.de) for researchers who meet the criteria for access to confidential data (please provide the manuscript title with your enquiry).

## Supplementary Materials

The following supporting information can be downloaded at: https://www.mdpi.com/article/10.3390/diagnostics13040661/s1, Figure S1: Miller’s knowledge pyramid; Figure S2: example direct observation of procedural skills (DOPS) test sheets: (**A**) examiner sheet; (**B**) participant’s task sheet; (**C**) scoring scheme; Table S1: characteristics of the participants (*n* = 76); Table S2: characteristics of the examiners (*n* = 10).

## References

[1] Bundesärztekammer [Federal Medical Association] (2021). (Muster-)Weiterbildungsordnung 2018 in der Fassung vom 26.06.2021.

[2] Welle R., Seufferlein T., Kratzer W. (2021). Current state of under- and postgraduate education in abdominal ultrasonography at German university hospitals. A panel study over 20 years. Z. Gastroenterol..

[3] Recker F., Blank V., Diederich H., Huckauf S., Lindner F., Minier M., Neubauer B., Wielandner A., Sachs A. (2014). Ultraschallausbildung an deutschsprachigen Universitäten: Wo stehen wir und wo soll es hingehen?. Ultraschall Med.—Eur. J. Ultrasound..

[4] Cantisani V., Jenssen C., Dietrich C.F., Ewertsen C., Piscaglia F. (2022). Clinical practice guidance and education in ultrasound: Evidence and experience are two sides of one coin!. Ultraschall Med..

[5] (2015). Sektion-Kopf-Hals D. Kurscurriculum der Sektion Kopf—Hals Degum.de: Deutsche Gesellschaft für Ultraschall in der Medizin. https://www.degum.de/fileadmin/dokumente/sektionen/kopf-hals/KPF_2015__Kurscurriculum_2015-05-18.pdf.

[6] Kassenärztliche Bundesvereinigung (KBV) (2021). Vereinbarung von Qualitätssicherungsmassnahmen nach § 135 Abs. 2 SGB V zur Ultraschalldiagnostik (Ultraschall-Vereinbarung) vom 31.10.2008 in der ab dem 01.10.2021 geltenden Fassung. https://www.kbv.de/media/sp/Ultraschallvereinbarung.pdf.

[7] Künzel J., Bozzato V., Ernst B.P., Bozzato A. (2022). Musterbefund zur sonografischen Diagnostik im Kopf-Hals-Bereich (Sektion Kopf-Hals der DEGUM). Ultraschall Med.—Eur. J. Ultrasound..

[8] Todsen T., Ewertsen C., Jenssen C., Evans R., Kuenzel J. (2022). Head and Neck Ultrasound—EFSUMB Training Recommendations for the Practice of Medical Ultrasound in Europe. Ultrasound Int. Open..

[9] Schuwirth L., van der Vleuten C. (2004). Merging views on assessment. Med. Educ..

[10] Ben-David M.F. (2000). The role of assessment in expanding professional horizons. Med. Teach..

[11] Miller G.E. (1990). The assessment of clinical skills/competence/performance. Acad. Med. J. Assoc. Am. Med. Coll..

[12] Harden R.M., Gleeson F.A. (1979). Assessment of clinical competence using an objective structured clinical examination (OSCE). Med. Educ..

[13] Norcini J.J., Blank L.L., Duffy F.D., Fortna G.S. (2003). The mini-CEX: A method for assessing clinical skills. Ann. Intern. Med..

[14] Heinzow H.S., Friederichs H., Lenz P., Schmedt A., Becker J.C., Hengst K., Marschall B., Domagk D. (2013). Teaching ultrasound in a curricular course according to certified EFSUMB standards during undergraduate medical education: A prospective study. BMC Med. Educ..

[15] Erfani Khanghahi M., Ebadi Fard Azar F. (2018). Direct observation of procedural skills (DOPS) evaluation method: Systematic review of evidence. Med. J. Islam Repub Iran..

[16] ul Haq I., Jamil B., Durrani M. (2018). Procedural skills. Prof. Med. J..

[17] Mohamadirizi S., Mardanian F., Torabi F. (2020). The effect of direct observation of procedural skills method on learning clinical skills of midwifery students of medical sciences. J. Educ. Health Promot..

[18] Höhne E., Recker F., Dietrich C.F., Schäfer V.S. (2022). Assessment Methods in Medical Ultrasound Education. Front. Med..

[19] Todsen T., Tolsgaard M.G., Olsen B.H., Henriksen B.M., Hillingsø J.G., Konge L., Jensen M.L., Ringsted C. (2015). Reliable and valid assessment of point-of-care ultrasonography. Ann Surg..

[20] Hofer M., Kamper L., Sadlo M., Sievers K., Heussen N. (2011). Evaluation of an OSCE assessment tool for abdominal ultrasound courses. Ultraschall Med..

[21] Tolsgaard M.G., Todsen T., Sorensen J.L., Ringsted C., Lorentzen T., Ottesen B., Tabor A. (2013). International multispecialty consensus on how to evaluate ultrasound competence: A Delphi consensus survey. PLoS ONE.

[22] Todsen T., Melchiors J., Charabi B., Henriksen B., Ringsted C., Konge L., von Buchwald C. (2018). Competency-based assessment in surgeon-performed head and neck ultrasonography: A validity study. Laryngoscope.

[23] Rehman I., Shah R., Kamal A., Ahmed M. (2019). Using DOPS (directly observed procedural skills) for pre call assessment of ultrasound proficiency of first year radiology residents: Development and initial analysis. Pak J. Radiol..

[24] Kara C.O., Mengi E., Tümkaya F., Topuz B., Ardıç F.N. (2018). Direct observation of procedural skills in otorhinolaryngology training. Turk. Arch. Otorhinolaryngol..

[25] Bansal M. (2019). Introduction of directly observed procedural skills (DOPS) as a part of competency-based medical education in otorhinolaryngology. Indian J. Otolaryngol. Head Neck Surg..

[26] Awad Z., Hayden L., Muthuswamy K., Ziprin P., Darzi A., Tolley N.S. (2014). Does direct observation of procedural skills reflect trainee’s progress in otolaryngology?. Clin. Otolaryngol..

[27] Weimer J.M., Rink M., Müller L., Arens C., Bozzato A., Künzel J. (2022). Sonographic diagnostics in the head and neck area, part 2—Transcervical sonography. Laryngorhinootologie.

[28] Streiner D.L., Norman G.R., Cairney J. (2015). Health Measurement Scales: A Practical Guide to Their Development and Use.

[29] Arbeitskreis “Lehrevaluation” im Fach Psychologie (Gläßer, E., Gollwitzer, M., Kranz, D., Meiniger, C., Schlotz, W., Schnell, T. & Voß, A.) in Zusammenarbeit mit dem Zentrum für Psychologische Diagnostik, Begutachtung und Evaluation (ZDiag). 2002. TRIL. Trierer Inventar zur Lehrevaluation [Verfahrensdokumentation, Fragebogen für je Weibliche und Männliche Dozierende]. In Leibniz-Institut für Psychologie (ZPID) (Hrsg.), Open Test Archive. Trier: ZPID. https://www.psycharchives.org/en/item/b6adcb29-8e57-45b0-91cb-f1931c0fb552.

[30] Pierre R.B., Wierenga A., Barton M., Branday J.M., Christie C.D.C. (2004). Student evaluation of an OSCE in paediatrics at the University of the West Indies, Jamaica. BMC Med. Educ..

[31] Weisser F.O., Dirks B., Georgieff M. (2004). Objective Structured Clinical Examination (OSCE): Eine neue Prüfungsform in der notfallmedizinischen Ausbildung. Notf Rett..

[32] Dabir S., Hoseinzadeh M., Mosaffa F., Hosseini B., Dahi M., Vosoughian M., Moshari M., Tabashi S., Dabbagh O. (2021). The effect of repeated direct observation of procedural skills (R-DOPS) assessment method on the clinical skills of anesthesiology residents. Anesthesiol. Pain Med..

[33] Jang T., Aubin C., Naunheim R. (2004). Minimum training for right upper quadrant ultrasonography. Am. J. Emerg. Med..

[34] Duanmu Y., Henwood P.C., Takhar S.S., Chan W., Rempell J.S., Liteplo A.S., Koskenoja V., Noble V.E., Kimberly H.H. (2019). Correlation of OSCE performance and point-of-care ultrasound scan numbers among a cohort of emergency medicine residents. Ultrasound J..

[35] DEGUM (2022). Prüfung Stufe II Anästhesiologie Deutsche Gesellschaft für Ultraschall in der Medizin. https://www.degum.de/fachgebiete/sektionen/anaesthesiologie/mehrstufenkonzept-zertifizierung/pruefung-stufe-ii-anae.html.

[36] Hochlehnert A., Schultz J.-H., Möltner A., Tımbıl S., Brass K., Jünger J. (2015). Electronic acquisition of OSCE performance using tablets. GMS Z. Med. Ausbild..

[37] Kuhn S., Frankenhauser S., Tolks D. (2018). Digital learning and teaching in medical education: Already there or still at the beginning?. Bundesgesundheitsblatt Gesundh. Gesundh..

