# Supplementary material for: Development and Integration of DOPS as Formative Tests in Head and Neck Ultrasound Education: Proof of Concept Study for Exploration of Perceptions
Source: Diagnostics (Basel). 2023 Feb 10;13(4):661. doi: 10.3390/diagnostics13040661 (PMC9954978; doi:10.3390/diagnostics13040661)
Supplement: Supplementary file 1 [file diagnostics-13-00661-s001.zip › diagnostics-2205392-supplementary.pdf]

Supplementary Table S1 Characteristics of the participants (n = 76)

Variable	n (%)
**Course format**	
Basic	57 (75.0)
Advanced	19 (25.0)
**Mean age** (year, IQR)	30 (27–32)
**Sex**	
Male	39 (51.3)
Female	37 (48.7)
**Medical specialty**	
Otorhinolaryngology	61 (80.3)
Oral and maxillofacial surgery	12 (15.8)
Internal medicine	2 (2.6)
Nuclear medicine	1 (1.3)
**Position**	
Resident	69 (90.8)
Specialist	7 (9.2)
**Previous ultrasound course**	
No	50 (65.8)
Yes	26 (34.2)
Course duration	
< 10 hours	4
10–20 hours	18
> 20 hours	5
**Self-performed ultrasound examinations**	
None	5 (6.6)
Less than 20	11 (14.5)
Between 20 and 50	19 (25.0)
Between 51 and 100	14 (18.4)
More than 100	27 (35.5)
Advanced course participants	15

Variable	n (%)
Basic course participants	12
**Pre-course self-assessment of competence**	Score – 7-point Likert scale
Ultrasound device handling	4
Ultrasound probe handling	3
Knowledge of ultrasound anatomy	3
Practical ultrasound competence	3

IQR, interquartile range.

Supplementary Table S2 Characteristics of the examiners (n = 10)

Variable	n (%)
**Sex**	
Male	9 (90.0)
Female	1 (10.0)
**Medical speciality**	
Otorhinolaryngology	5 (50.0)
Neurosurgery	1 (10.0)
General surgery	1 (10.0)
Internal medicine	2 (20.0)
Neurology	1 (10.0)
**Position**	
Specialist	6 (60.0)
With DEGUM level II or III certification	4
Resident	4 (40.0)
**Previous teaching experience**	
Yes	10 (100.0)
No	0 (0.0)

Supplementary Figure S1 Miller´s knowledge pyramid

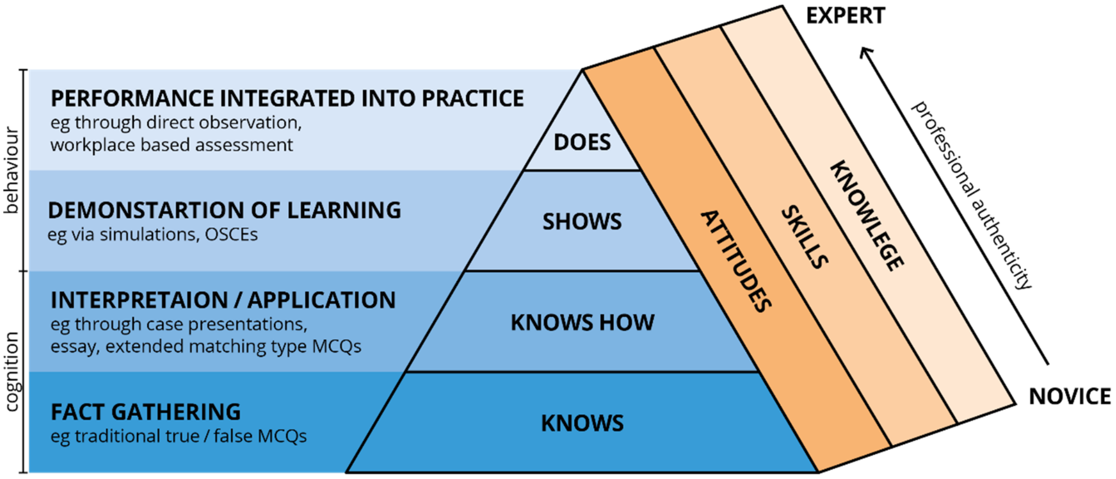

Based on work by Miller GE, The Assessment of Clinical Skills / Competence / Performance: Acad. Med. 1990; 65(9); 63-67
Adapted by Drs. R. Mehay & R. Bums. UK (Jan 2009)

Supplementary Figure S2 Example direct observation of procedural skills (DOPS) test sheets: A

Examiner sheet; **B** Participant´s task sheet; **C** Scoring scheme

A

HEAD-NECK-DOPS NR. 3: TOPIC "NECK – FLOOR OF MOUTH" – EXAMINER SHEET

UNIVERSITÄTSmedizin. RFLK | Rudolf Frey Lernklinik MAINZ — UKR Universitätsklinikum Regensburg

AUDIT OBJECTIVE:

1) The examinee masters **guiding** the patient **through the examination**, in an appropriate manner appropriate to it

2) The examinee masters the **handling of the transducer** regarding:
· Orientation · Positioning · Connection · adequate image setup and zoom factor

3) The examinee masters carrying out the **examination** in a correct way with regard to:
- …**scanning of the floor of mouth including the tounge**
- …**measurement of the sublingual gland comparing both sides**
- …**explanation of the frozen standard section image "transverse view of the floor of mouth"**
- …**storing or printing the image**
- …**the overall performance**

CASE VIGNETTE: You are the doctor in charge at a polyclinic. A patient presents with unclear dysphagia and pain in swallowing. You would like to carry out an ultrasound examination on your patient.

TASK 1: Guide your patient through the **examination** in an appropriate manner (max. 6 points).

TASK 2: **Scan** the **floor of mouth including the tounge** completely **in both planes**. Initially, please present the **transverse default plane** (max. 16 points) and identify the following **anatomical structures** (max. 4 points):
· Mylohyoid muscle · Tongue
· Digastric muscle · Sublingual gland

TASK 3: **Mark** the **sublingual gland** and determine the size of the glands comparing both sides (double image mode if applicable) (max. 6 points).

TASK 4: **Store or print** an image (max. 1 point).

EXAMINER INFORMATION: **Time allowance 10 minutes: 8 minutes of assessment, 2 minutes of feedback!** Please sum up the overall score after the assessment and **do not hand out the exam paper**. The **exam paper** contains three rating levels per task:

Green	Yellow	Red
The examinee completed the task in a correct and error-free way.	The examinee completed the task under difficulties / was reliant on the examiners assistance.	Despite the examiners assistance, the examinee was not able to carry out the task adequately.

B

HEAD-NECK-DOPS NR. 3: TOPIC "NECK – FLOOR OF MOUTH" – EXAMINEE SHEET

UNIVERSITÄTSmedizin. RFLK | Rudolf Frey Lernklinik MAINZ — UKR Universitätsklinikum Regensburg

CASE VIGNETTE: You are the doctor in charge at a polyclinic. A patient presents with unclear dysphagia and pain in swallowing. You would like to carry out an ultrasound examination on your patient.

TASK 1: Guide your patient through the **examination** in an appropriate manner.

TASK 2: **Scan** the **floor of mouth including the tounge** completely **in both planes**. Initially, please present the **transverse default plane** and identify the following **anatomical structures**:

· Mylohyoid muscle · Tongue
· Digastric muscle · Sublingual gland

TASK 3: **Mark** the **sublingual gland** and determine the size of the glands comparing both sides (double image mode if applicable).

TASK 4: **Store or print** an image.

C

HEAD-NECK-DOPS NR. 3: TOPIC „NECK – FLOOR OF MOUTH" – EVALUATION SHEET

UNIVERSITÄTSmedizin. RFLK | Rudolf Frey Lernklinik MAINZ — UKR Universitätsklinikum Regensburg

EXAMINER: ____________ **EXAMINEE:** ____________

TASK 1: Guide your patient through the **examination** in an appropriate manner.

Patient guidance (max. 6 points)

The examinee mentions: **"Please overstretch your head and slightly tilt it to the side"**	2 ☐	1 ☐	0 ☐
The examinee mentions: **"Please remove any clothes from your neck area"**	2 ☐	1 ☐	0 ☐
The examinee mentions: **"Putting on gloves and handing paper towels to the patient."**	2 ☐	1 ☐	0 ☐

TASK 2: **Scan** the **floor of mouth including the tounge** completely **in both planes**. Initially, please present the **transverse default plane** and identify the following **anatomical structures**: **Mylohyoid muscle, Digastric muscle, Tongue, Sublingual gland**

Handling of the transducer (max. 8 points)

**Orientation**	
Correct or immediately corrects after disconnecting the left / cranial part of the transducer	2 ☐
Corrects after initial difficulties or upon request	1 ☐
Relies on the examiners manual help to find the correct orientation	0 ☐
**Positioning**	
Correct or immediately transfers to the correct position after starting elsewhere	2 ☐
Corrects after initial difficulties or upon request	1 ☐
Relies on the examiners manual help to find the correct positioning	0 ☐
**Connection**	
Connects the transducer adequately using enough ultrasound gel / adjusting the pressure	2 ☐
Corrects after initial difficulties or upon request	1 ☐
inadequate pressure / not enough ultrasound gel / not able to connect the transducer to the patient without manual help	0 ☐
**adequate image setup and zoom factor**	
Independently sets up an adequate image regarding gain, depth, frequency and focus settings	2 ☐
Corrects after initial difficulties or upon request	1 ☐
Relies on the examiners manual help to obtain an appropriate image setup	0 ☐

Scanning performance (max. 8 points)

**Transverse scanning**	
Scans floor of mouth and tongue completely in transversal plane in a reasonable time frame	3 ☐
Does not scan every structure entirely (e.g. forgets to increase the depth to examine the tongue) or inadequate time frame	2 ☐
Relies on manual help to scan the area or only scans marginal parts of the floor of mouth	1 ☐
No adequate examination possible despite the examiners manual help	0 ☐
**Sagittal scanning**	
Scans floor of mouth and tongue completely in sagittal plane in a reasonable time frame	3 ☐
Does not scan every structure entirely (e.g. forgets to increase the depth to examine the tongue) or inadequate time frame	2 ☐
Relies on manual help to scan the area or only scans marginal parts of the floor of mouth	1 ☐
No adequate examination possible despite the examiners manual help	0 ☐

TASK 3: **Mark** the **sublingual gland** and determine the size of the glands comparing both sides (double image mode if applicable).

Measurement (max. 6 points)

Depicts both glands correctly applying double image mode and uses correct endpoints for the measurement	6 ☐
Inadequate image setting of both glands or wrong endpoints / corrects upon the examiners verbal request	4 ☐
Relies on manual help to carry out the image setting and measurements.	2 ☐
No adequate image setup / measurements despite the examiners manual help.	0 ☐

TASK 2+4: **Explain** and **store or print** an image.

Explanation and image documentation: Points out and identifies the following anatomical structures correctly **(max. 5 points)**

Mylohyoid muscle	1 ☐	Tongue	1 ☐
Digastric muscle	1 ☐	Sublingual gland	1 ☐
Store or print the image			1 ☐

OVERALL PERFORMANCE: The overall performance is rated with: (max. 6 points – circle the number)

1 – 2 – 3 – 4 – 5 – 6

OVERALL SCORE: ________ / 37

authors: J. Weimer, A. Hollinderbäumer, M. Rink, K. Dirks, J. Künzel
